# Alcohol-Preferring Rats Show Goal Oriented Behaviour to Food Incentives but Are Neither Sign-Trackers Nor Impulsive

**DOI:** 10.1371/journal.pone.0131016

**Published:** 2015-06-22

**Authors:** Yolanda Peña-Oliver, Chiara Giuliano, Daina Economidou, Charles R. Goodlett, Trevor W. Robbins, Jeffrey W. Dalley, Barry J. Everitt

**Affiliations:** 1 Department of Psychology and Behavioural and Clinical Neuroscience Institute, University of Cambridge, Downing St, Cambridge, United Kingdom; 2 Department of Psychology, Indiana University–Purdue University Indianapolis, Indianapolis, Indiana, United States of America; 3 Department of Psychiatry, University of Cambridge, Addenbrooke’s Hospital, Cambridge, United Kingdom; VU university medical center, NETHERLANDS

## Abstract

Drug addiction is often associated with impulsivity and altered behavioural responses to both primary and conditioned rewards. Here we investigated whether selectively bred alcohol-preferring (P) and alcohol-nonpreferring (NP) rats show differential levels of impulsivity and conditioned behavioural responses to food incentives. P and NP rats were assessed for impulsivity in the 5-choice serial reaction time task (5-CSRTT), a widely used translational task in humans and other animals, as well as Pavlovian conditioned approach to measure sign- and goal-tracking behaviour. Drug-naïve P and NP rats showed similar levels of impulsivity on the 5-CSRTT, assessed by the number of premature, anticipatory responses, even when the waiting interval to respond was increased. However, unlike NP rats, P rats were faster to enter the food magazine and spent more time in this area. In addition, P rats showed higher levels of goal-tracking responses than NP rats, as measured by the number of magazine nose-pokes during the presentation of a food conditioned stimulus. By contrast, NP showed higher levels of sign-tracking behaviour than P rats. Following a 4-week exposure to intermittent alcohol we confirmed that P rats had a marked preference for, and consumed more alcohol than, NP rats, but were not more impulsive when re-tested in the 5-CSRTT. These findings indicate that high alcohol preferring and drinking P rats are neither intrinsically impulsive nor do they exhibit impulsivity after exposure to alcohol. However, P rats do show increased goal-directed behaviour to food incentives and this may be associated with their strong preference for alcohol.

## Introduction

Impulsivity, the tendency to exhibit unduly premature or risky behaviour, has been associated with addiction to several drugs of abuse including alcohol but the directionality of this relationship is not entirely clear. Thus, impulsivity might be a vulnerability factor to develop alcoholism or might appear as a consequence of repeated alcohol use and withdrawal. Demonstration of causal relationships in human studies is difficult; inferences must be made from retrospective data, and impulsivity in individuals using or having used alcohol may reflect an effect of the drug. Nevertheless, longitudinal studies performed with subjects with a family history of alcoholism seem to suggest a positive relationship between higher levels of impulsivity and the development of alcohol abuse disorders [1–3].

Animal studies have the advantage of assessing impulsivity in alcohol-naïve subjects with a known genetic background and propensity to drink, allowing the assessment of impulsivity both before and after exposure to alcohol. Several rodent strains have been genetically bred to display marked differences in alcohol related behaviours. Thus, alcohol-preferring (P) and alcohol-nonpreferring (NP) rats have been bi-directionally bred for their consumption of ethanol in a 2-bottle choice drinking procedure. Alcohol-preferring P rats show high levels of ethanol preference over water when given free access to both solutions, unlike NP rats which show very low levels of voluntary ethanol drinking under the same conditions [4,5].

We initially hypothesised that P rats might show high levels of impulsivity in the 5-choice serial reaction time task (5-CSRTT) prior to any opportunity to drink alcohol, in comparison with NP rats. This hypothesis was based on the association between alcohol abuse and heightened impulsivity in humans [6,7], and the finding that impulsivity in the 5-CSRTT is positively correlated with ethanol drinking in several strains of mice [8–10] and an analogous task in humans [8,11].

Motor impulsivity, the tendency to act rashly, reflecting an inability to restrain inappropriate or prepotent responses, which might lead to negative consequences, has been shown strongly to predict the escalation of cocaine intake and the development of compulsive cocaine seeking in rats [12], as well as nicotine self-administration [13] but did not predict loss of control over heroin self-administration in rats [14] suggesting that impulsivity does not predict the tendency to self-administer or lose control over all addictive drugs.

Although not primarily studied in the 5-CSRTT, P and NP rats have been assessed for impulsivity using other measures. Previous studies using a differential reinforcement of low-rate responding (DRL) task suggested no marked differences in motor impulsivity between P and NP rats, nor between the high alcohol drinking (HAD1) and low alcohol drinking (LAD1) lines, that were also bred for their differential levels of alcohol drinking and preference [15], and even suggested that P rats showed reduced motor impulsivity compared with Wistar rats in a DRL-10s procedure [16]. Nevertheless, in paradigms of choice impulsivity, e.g. delay-discounting tasks, in which impulsive individuals show a preference for options leading to immediate gratification, but are detrimental in the long term, versus options associated with delayed benefits but that provide a more advantageous outcome in the long run, high alcohol-preferring mice [17] and rats [18,19] have shown steeper discounting and hence are more impulsive than the low preferring strains, although this finding has not always been replicated [10,20,21].

To further characterise P and NP rats we investigated whether they were differentially impaired in the acquisition of appetitive Pavlovian conditioning since this has been shown to predict measures of stimulant self-administration and the incentive salience of drug-associated stimuli [22,23]. In this task, some subjects will approach the location in which the reward is delivered, showing a goal-tracking response while others develop an approach response towards the CS predictive of reward delivery and interact with it (sign-trackers). Thus, it has been suggested that differences in this apparent attribution of incentive salience to discrete cues predictive of reward in sign-tracking rats might be associated with impulsivity [24–26] and may predict vulnerability to drug use [22,23,25,27].

In the present study we also assessed P and NP rats in an intermittent 2-bottle choice drinking procedure prior to re-testing in the 5-CSRTT, in order both to validate and replicate the drinking phenotype observed in these lines in our laboratory and to investigate if chronic access to alcohol in the P rats would impact on our measure of impulsivity.

## Material and Methods

### Subjects

Fifteen male P rats and thirteen male NP rats of 4–5 weeks of age were provided by Indiana University Medical Centre (Indianapolis, IN) and accustomed to the animal facility for 3 weeks before the start of the experiments. Rats were housed in groups of 2–4 per cage up until the two-bottle choice procedure whereupon they were singly-housed. Throughout the study animals were maintained in a temperature- and humidity-controlled room (22°C) under a reversed light/dark cycle (white lights off/red lights on from 7:00 a.m. to 7:00 p.m.). When body weights had reached at least 340 g a food restricted diet (18 g chow/subject/day) was imposed to maintain weights at 85–90% of free-feeding weights. This was implemented for the entire duration of the study, except where otherwise specified. Water was available *ad libitum*. All experimental procedures were carried out in accordance with the regulatory requirement of the UK Animal (Scientific Procedures) Act of 1986 and Council Directive 2010/63EU of the European Parliament, and were approved by the University of Cambridge Institutional Animal Care and Use committee.

### Behavioural apparatus

The 5-CSRTT apparatus consisted of eight rat operant chambers (Med Associates Inc., St. Albans, Vermont, USA). Each chamber was housed in a sound-attenuating outer cabinet, with a ventilator fan providing a constant low-level background noise. The rear wall of the chamber was curved and contained 5 apertures fitted with infrared detectors to detect nose-poke responses. The apertures were illuminated by a yellow stimulus light located inside each aperture. On the opposite side of the chamber, a magazine connected to a food dispenser allowed the automatic delivery of 45 mg of reward pellets (Noyes dustless pellets, Research Diets, UK). Subjects gained access to the food magazine by pushing a panel monitored by a micro-switch. A house-light was located at the top of the wall above the food magazine.

Twelve operant chambers were used for the Pavlovian conditioning experiments, different from the ones used for the 5-CSRTT, and located in a different room (Med Associates Inc., St. Albans, Vermont, USA). These were equipped with two retractable levers, a pellet receptacle and dispenser between the levers on the same wall, a cue light above each lever and a white house light. Active and inactive levers were counterbalanced between the left and right sides of the behavioural chamber.

### Behavioural procedures on the 5-choice task

On two consecutive days before the start of the behavioural testing, rats were given 20 g of reward pellets in their home-cage prior to their daily feeding, in order to habituate them to the reinforcer. During the first two sessions of training animals were habituated in the boxes for 30 min. During these sessions several pellets were placed in each of the five apertures and the magazine in order to facilitate exploration of the chamber and nose-poking behaviour.

The 5-CSRTT training began with the illumination of the house-light and free delivery of a food pellet in the magazine accompanied by the illumination of the food magazine, which signalled reinforcer availability. When the animal nose-poked in the magazine to collect the pellet, the first trial was initiated. After a fixed interval (ITI), one of the stimulus lights in the holes was turned on for a brief time and the animal was required to nose-poke within a certain period of time (limited hold, LH) into the correct hole in order to obtain the reinforcer. After a correct response, the animal was required to nose-poke into the food magazine to collect the reinforcer and to initiate the next trial. An incorrect response occurred when the animal made a response in a different hole to the one that had been illuminated, and this was followed by a time out period (TO) during which time the lights were turned off for 5 s. Responses made into the holes during this period restarted the TO. An error of omission occurred when the animal failed to respond into any of the holes after the completion of the LH and was also followed by a TO. Any response into the holes during the ITI (i.e., before the stimulus light had been presented) was registered as a premature response and was followed by a TO. After a TO period, the next trial was restarted by a nose-poke into the magazine. Perseverative responses into any of the response holes after a correct response but before the collection of food were registered but had no programmed consequences.

At the beginning of training, the stimulus duration (SD) was set to 30 s and the ITI was set to 2s. After achieving performance criteria (>50 correct trials, >75% accuracy,<25% omissions), the stimulus duration was reduced in the following pattern: 30, 20, 10, 5, 2.5, 1.8, 1.4, 1.2, 1, 0.9, 0.8, 0.7, 0.6, 0.5 (baseline) and the LH and ITI were set at 5 s. Testing was carried out daily (5–6 days/week), and the session lasted for 100 trials or 30 min, whichever came first. After 2 weeks of stable responding, rats were tested under conditions designed to increase premature responding. During this 60 min session the ITI was increased to 7 s to facilitate the likelihood of a premature response [28]. On the day following the long ITI challenge session rats were tested under the training ITI of 5 s. Levels of impulsivity were evaluated in P and NP rats before and after access to ethanol. Animals were not food restricted for the duration of the intermittent access two bottle choice procedure.

The behavioural variables used for the analysis of 5-CSRTT performance were (i) accuracy: correct responses/(correct responses + incorrect responses) x 100; (ii) omissions: total omissions/(correct responses + incorrect responses + omissions) x 100; (iii) premature responses: premature responses/(correct responses + incorrect responses + omissions) x 100; (iv) correct response latency: latency to nose-poke into the correct hole after the onset of the stimulus (s); (v) magazine latency: latency to collect the reward after a correct response (s); (vi) time in magazine: time spent nose-poking in the food magazine (s).

### Pavlovian conditioned approach

Our procedure was adapted from that described by [29]. Prior to training, rats were familiarised with chocolate flavoured food pellets (45 mg precision food pellets, Research Diets, UK) in their home-cage for two consecutive days. The following day, rats were placed into the operant chambers for a pre-training session. Here 50 food pellets were delivered in the food-magazine following a variable interval 30-s schedule (pellet delivered between 0-60s). After this pre-training session the task was implemented during 5 consecutive daily sessions. For each session, a trial consisted of the insertion of a lever (CS) into the chamber for 8 s on a VI 55-s schedule. The retraction of the lever was followed by the delivery of a food pellet in the food magazine. Each session consisted of 25 trials (CS-US pairings), and pellet delivery was independent of the behaviour of the animal. The measures recorded were the total number of lever presses and total number and time spent nose-poking into the food magazine during the presentation of the CS (lever).

### Ethanol preference test

Rats were individually housed and provided with 24 h intermittent access to two bottles containing tap water or 10% v/v ethanol for a period of 4 weeks. The two bottles were available for 24 hours on Monday, Wednesday and Friday, and bottles were weighed prior to and after each 24 h period. Solutions were prepared and changed weekly and provided at room temperature. The positions of the bottles were alternated every day to prevent side preferences. Ethanol consumption (g alcohol/kg animal body weight/day) and ethanol preference over the total fluid intake (%) were the main dependent variables.

### Statistical analyses

Inferential statistics were carried out using the ‘Statistical Package for Social Sciences’ (SPSS, version 20.0). Behavioural data on the 5-CSRTT were analysed using repeated measures ANOVA with group (2 levels: P vs NP rats) as the between subject’s factor and alcohol exposure (2 levels: pre and post alcohol) and session (3 levels: baseline1, long ITI, baseline2) as within subject’s factors. Significant interactions were further analysed using one-way ANOVAs and paired samples t-tests. Total number of sessions to achieve criteria of performance in the 5-CSRTT was analysed by a non-parametric Kruskal-Wallis test. Data from the Pavlovian conditioned approach task, ethanol preference and intake were analysed with repeated measures ANOVA with group and session as between and within subject’s factors, respectively. For the session by session comparisons of ethanol preference and intake, significant p values were adjusted after Bonferroni corrections. Where sphericity assumptions were violated, the Huynh Feldt correction was applied and the epsilon value is reported. Pearson’s correlation coefficient r was used to determine the relationships between impulsivity, goal- and sign-tracking and the time spent in magazine in the 5-CSRTT.

## Results

### 5-CSRTT

**Fig 1:**
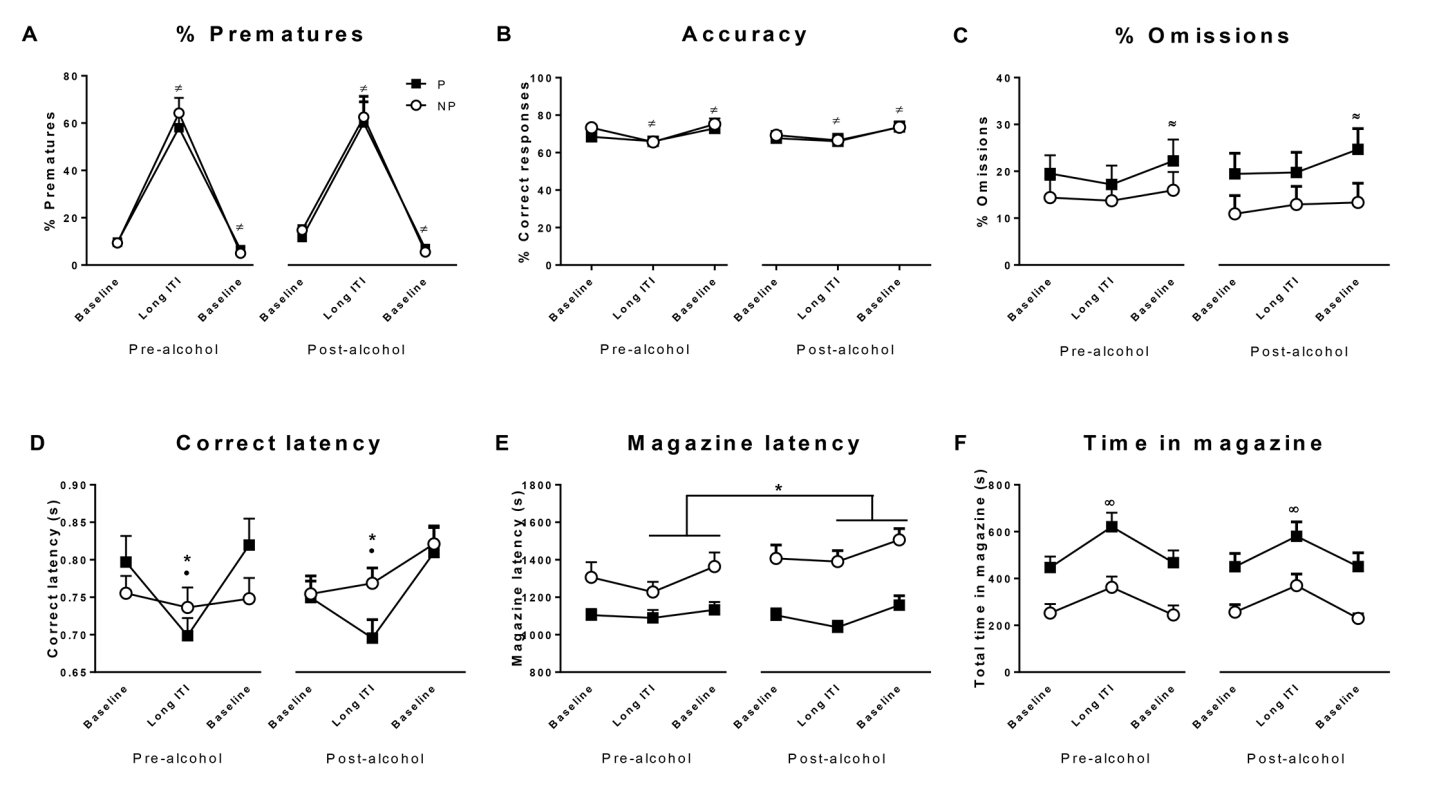
5-CSRTT. Performance of alcohol-preferring rats (P, closed squares) and alcohol-non-preferring rats (NP, open circles) in the 5-CSRTT, during baseline sessions (ITI = 5s) and during the long ITI challenge session (ITI = 7s) before and after the 4-week alcohol intake. Data are means ± SE of % premature responding (a), attentional accuracy (b), % omissions (c), correct response latency (d), food magazine latency (e) and time spend nose-poking in the food magazine (f). All p<0.05: (≠) vs baseline A, (σ) vs previous sessions; (*) significant difference between P and NP rats in the same session; (•) vs baseline A in P rats, (∞) vs baseline A and B; (x) versus pre-alcohol sessions in NP rats.

There were no statistical differences in the number of sessions required to achieve baseline performance criteria (NP: 33.54 ± 1.2, P: 31.67 ± 1.8, χ^2^_(1)_ = .308, n.s.). P and NP rats showed no significant difference in premature responses during the baseline and long ITI challenge sessions (group x session interaction: F_2,52_ = .325, n.s; group: F_1,26_ = .138, n.s; Fig 1A). Both P and NP rats showed increased premature responding during the long ITI session (session: F_2,52_ = 141.981, p<0.001, ε = .566) but this increase was no different between groups nor was it significantly modulated by 4-weeks of alcohol exposure, despite P rats consuming significantly more ethanol than NP rats (see below). Attentional accuracy (Fig 1B) and omissions (Fig 1C) were also generally unaffected by phenotypic status and ethanol exposure. Thus, although accuracy decreased during the long ITI session and increased during the following baseline session these changes were no different between P and NP rats. Omissions increased significantly in both groups during the final baseline session but this change was small and not different between P and NP rats. However, correct response latencies were differentially affected in P and NP rats (group x session interaction: F_2,52_ = 5.14, p<0.01, Fig 1D) with ethanol-exposed P rats being significantly faster to respond than NP rats during the long ITI session (p<0.05).

Alcohol-preferring P rats also showed enhanced magazine-directed behaviour compared with NP rats. Our analysis revealed a significant interaction between group and alcohol exposure (F_1,26_ = 6.267, p<0.05, Fig 1E) and a significant main effect of group (F_1,26_ = 18.02, p<0.001) showing that P rats were faster to approach and enter the food magazine than NP rats. P rats also spent longer nose poking in the food magazine compared with NP rats (group: F_1,26_ = 11.86, p<0.01, Fig 1F). Alcohol exposure had no significant effect on magazine latencies in P rats (see Fig 1E). However, in NP rats, magazine latencies significantly increased following intermittent alcohol intake. Finally, both groups showed an increase in time spent in the magazine during the long ITI challenge session (F_2,52_ = 50.9, p<0.001, ε = 0.612, Fig 1F) regardless of their alcohol experience (all F values < 0.8, n.s.).

### Pavlovian conditioned approach

**Fig 2:**
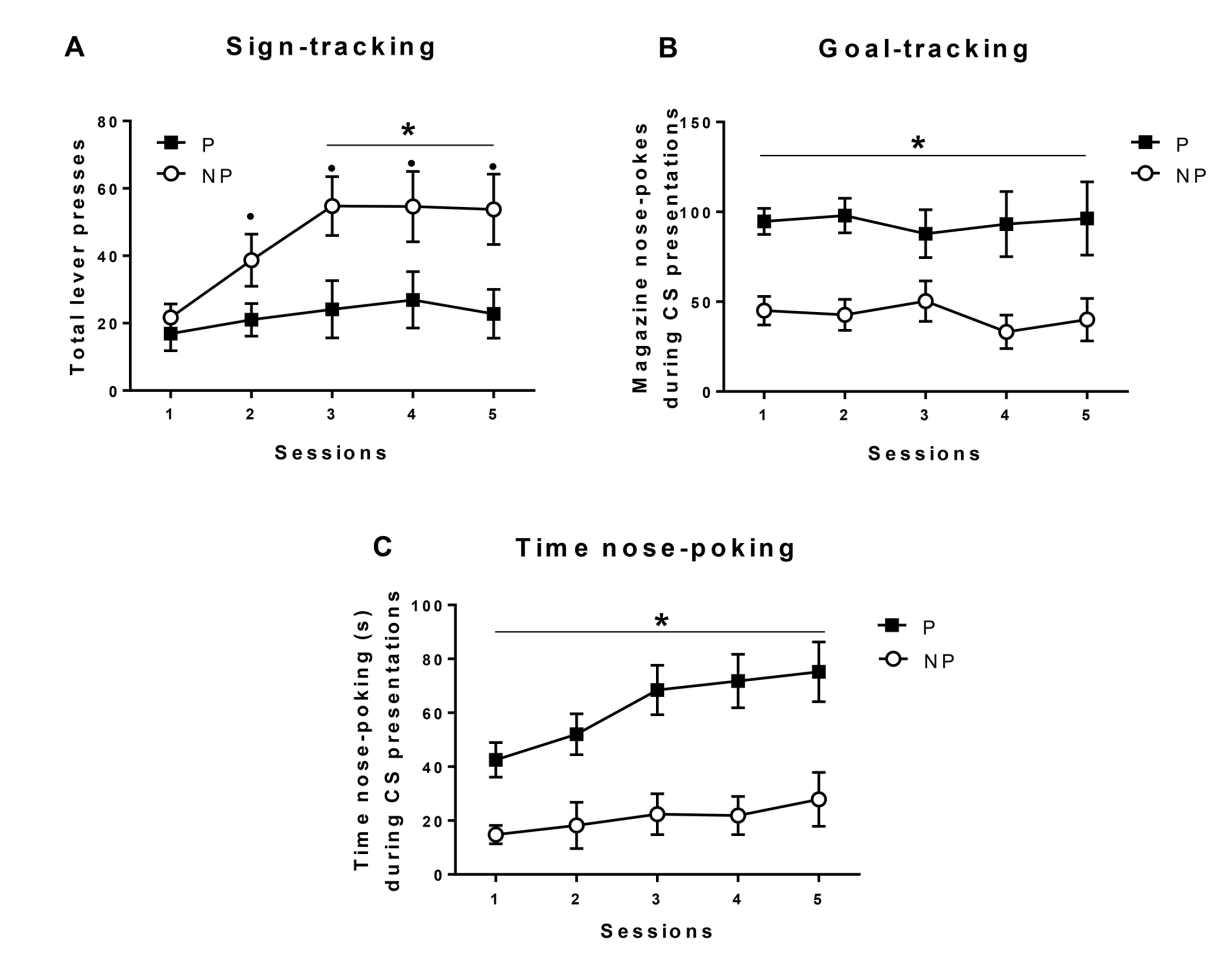
Pavlovian conditioned approach. Acquisition of Pavlovian conditioned approach in alcohol-preferring rats (P, closed squares) and alcohol-non-preferring rats (NP, open circles). Data are means ± SE of total lever presses (sign-tracking responses, a), total magazine nose-pokes during the CS-lever presentations (goal-tracking responses, b), and total time spent nose-poking during the CS-lever presentations (c). All p<0.05: (*) significant group differences, (•) versus session 1 in NP rats.

The above findings indicate that P rats are faster to enter the food magazine and spend more time in its vicinity. To investigate the hypothesis that P rats show altered motivational responses to the primary reward itself we compared P and NP rats in the acquisition of approach responses to the primary reward (goal-tracking) and a conditioned stimulus predictive of food reward (sign-tracking). Alcohol-preferring P rats showed diminished sign-tracking responses compared with NP rats; the latter progressively increasing their lever-press responses each session (group x session interaction: F_4,104_ = 3.86, p<0.05; group: F_1,26_ = 5.29, p<0.05, Fig 2A). In marked contrast, P rats showed high levels of goal-directed responses in the food magazine compared with NP rats (main effect of group, F_1,26_ = 10.30, p<0.01, Fig 2B). There were no session or session x group interactions (all F values <0.855, n.s.). P rats also spent more time nose-poking in the magazine in comparison with NP rats (main effect of group, F_1,26_ = 15.775, p<0.01, Fig 2C), and time spent nose-poking increased across sessions in both groups (session effect: F_4,104_ = 7.22, p<0.01). Although the session x group interaction did not reach significance, the increase in time spent nose-poking across sessions tended to be more pronounced in P rats.

### Ethanol preference and intake

**Fig 3:**
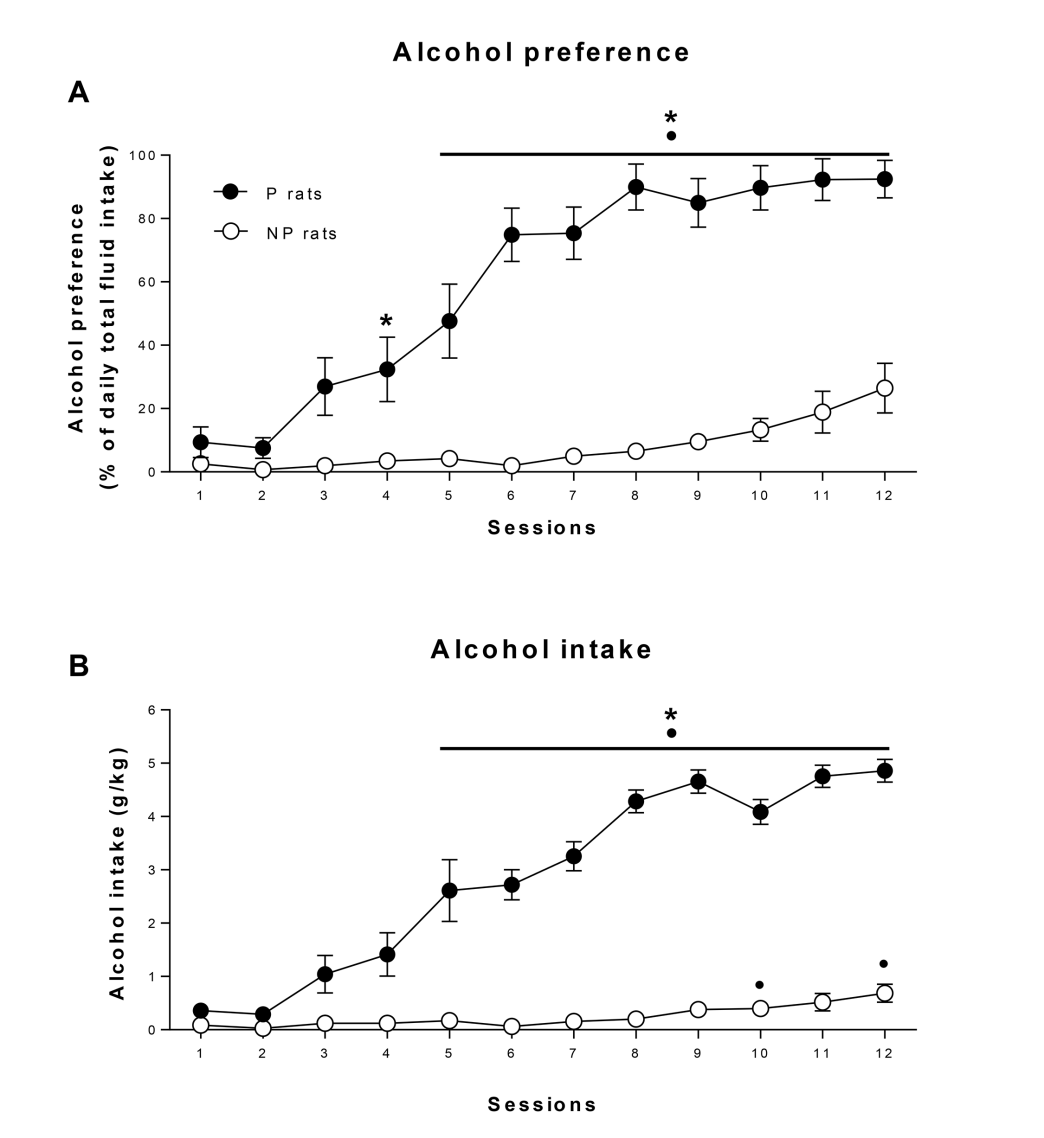
Intermittent access two-bottle choice procedure. Data represent the mean ± SE of alcohol preference (a) and alcohol intake (g/kg/day) (b) during the 12 sessions of intermittent access to alcohol in alcohol-preferring rats (P, closed squares) and alcohol-non-preferring rats (NP, open circles). All p<0.05 (adjusted after Bonferroni correction): (*) significant group differences, (•) vs session 1.

Fig 3 shows the total alcohol intake and preference for P and NP rats during the 12 sessions of intermittent access to alcohol and water in the home cage. P rats consumed significantly more alcohol than NP rats (group x session interaction: F_11,286_ = 36.78, p<0.001, ε = 0.32; group: F_1,26_ = 143.63, p<0.001, Fig 3A) and showed a significantly higher preference for alcohol (group x session interaction: F_11,286_ = 31.76, p<0.001, ε = 0.33, group: F_1,26_ = 189.94, p<0.001, Fig 3B). P rats consumed approximately 5 g alcohol/kg/day during the final 5 sessions whereas NP rats consumed less than 1g alcohol/kg/day over a similar period.

### Dimensional analysis

**Fig 4:**
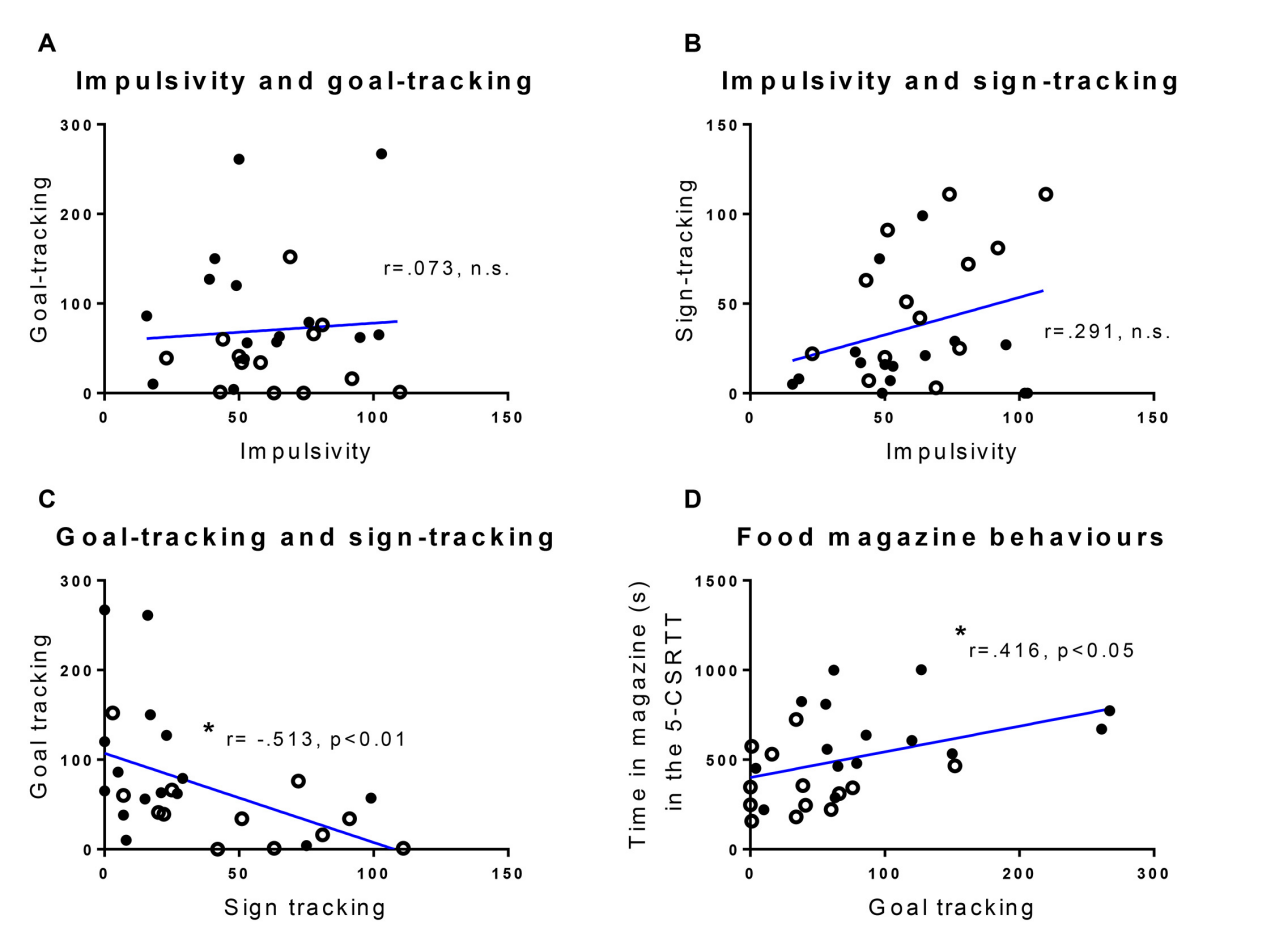
Correlations between main variables. Correlational analysis of impulsive responses during the first long ITI session in the 5-CSRTT and the data from the 5^th^ session of the Pavlovian conditioned approach task: Impulsivity and goal-tracking responses (number of magazine nose-pokes) (a); impulsive responses and sign-tracking responses (number of lever presses) (b); goal-tracking and sign-tracking responses (c); goal-tracking responses and time spent nose-poking in the food magazine during the first long ITI session in the 5-CSRTT) (d). P(closed circles), NP (open circles).

Fig 4 shows the relationships between impulsivity and magazine latencies in the 5-CSRTT and goal- versus sign-tracking behaviour in the Pavlovian conditioned approach task. Impulsivity was not correlated with either goal- or sign-tracking behaviour (p < 0.71, n.s, Fig 4A and 4B). Nevertheless, goal- and sign-tracking behaviour was negatively correlated (r = -0.51, p<0.01, Fig 4C) indicating, unsurprisingly, that high sign-tracking behaviour is associated with low levels of responding in the food magazine during the presentation of the CS (lever). Animals that spent longer nose-poking in the food magazine during the 5-CSRTT also showed high levels of goal-tracking behaviour in the Pavlovian conditioned approach task (r = 0.416, p<0.05, Fig 4D).

## Discussion

The main findings of this study confirm the greater preference and consumption of alcohol in selectively bred alcohol-preferring P rats compared with alcohol non-preferring NP rats. Despite this clear vulnerability, however, P rats were not more impulsive than NP rats either when assessed before or after a four week period of intermittent alcohol exposure. However, P rats showed faster response latencies to collect food reward, spent more time nose-poking in the food magazine in the 5-CSRTT, and showed increased goal-tracking responses in an appetitive Pavlovian conditioning task compared with NP rats. These findings demonstrate that vulnerability to alcohol intake is not predicted by increased levels of motor impulsivity on the 5-CSRTT, unlike psychostimulants [13,28] but instead by increased goal-directed behaviour to food incentives.

P and NP rats showed similar levels of premature responding in the 5-CSRTT even when the waiting interval to respond was increased. Although we initially hypothesised that P rats might show high levels of impulsivity compared with NP rats the existing literature regarding the evaluation of impulsivity in these two selected lines is scarce and inconsistent. In a study by Steinmez and colleagues [15] P, NP and Wistar rats were tested in a differential reinforcement of low-rate responding (DRL) task. This task evaluates motor impulsivity and is analogous to the construct of waiting impulsivity in the 5-CSRTT in that rats must withhold responding until a certain time interval has elapsed. P rats showed similar levels of lever pressing behaviour compared with NP and Wistar rats during a DRL-15s schedule suggesting similar levels of motor impulsivity between P and NP rats under these conditions [15]. In an earlier study, P rats were unimpaired in a similar DRL task compared with Wistar rats, with some evidence even of reduced impulsivity under some schedules [16]. These findings collectively indicate that alcohol-preferring P rats do not have an associated high level of motor impulsivity on the 5-CSRTT or DRL tasks and that impulsivity is not a factor predisposing them to drink alcohol.

However, other studies have reported an association between elevated levels of motor impulsivity and high alcohol drinking in several rodent lines not specifically bred for levels of ethanol drinking and preference. For instance, alcohol-naive C57BL/6J mice, which naturally show high levels of alcohol preference, also show higher levels of impulsivity in the 5-CSRTT compared with DBA2/J mice, a naturally alcohol-avoiding strain [8]. Furthermore, the BXD16 recombinant inbred mouse strain, characterised by an impulsive/inattentive phenotype in the 5-CSRTT also showed higher motivation to obtain an alcohol solution and enhanced cue-induced relapse of alcohol seeking [9].

An elevated alcohol drinking phenotype is not always accompanied by increased motor impulsivity. Thus, Wilhelm and Mitchell [20] studied selectively bred Sardinian alcohol-preferring (sP) and non-preferring (sNP) rats, as well as alko alcohol (AA) and alko non-alcohol (ANA) rats, in a delay discounting task. No differences in choice impulsivity were found between any of the lines tested, showing that genetic models of high alcohol consumption are not always accompanied by increased impulsivity [see also 10 for similar results in mice]. Conversely, in a previous study from the same authors, high alcohol drinking rats (HAD) showed higher levels of choice impulsivity than low alcohol drinking rats (LAD) [19], and a similar result was found in mice [17]. Overall, the results indicate that impulsivity may be a predictor of alcohol drinking in certain models but not in others. This is consistent with recent major prospective studies of ethanol drinking in human adolescents, which point that alcoholism has multiple aetiologies [30].

In a recent study two factors were assessed in relation to the high drinking phenotype: levels of consumption and operant seeking responses to obtain alcohol [18]. This study compared delay-discounting impulsivity in alcohol-preferring rats (P), which display high levels of both ethanol seeking and consumption, with high alcohol drinking rats (HAD2), that display moderate alcohol seeking but high consumption, and Long Evans rats (LE) that exhibit moderate alcohol seeking and consumption. The main findings indicated that only the high-seeking P rats showed high levels of delay discounting. Surprisingly, the high drinking HAD2 rats did not show elevated impulsivity, suggesting that choice impulsivity may be a vulnerability marker for high alcohol seeking rather than alcohol consumption. These results, together with our current data show the importance of taking into account the dimension of impulsivity being measured when seeking associations with elevated levels of alcohol seeking or consumption, since although choice impulsivity and motor impulsivity can be present in the same cohort of animals [31], these two impulsivity subtypes are neurally dissociable [32,33]. Furthermore, several studies have shown that choice and motor impulsivity are not correlated within the same population of animals [34] and a cross-translational study has shown no correlation between motor and choice impulsivity in rats and humans [35], indicating that some strains/individuals might show higher choice impulsivity and, at the same time, reduced motor impulsivity.

A possible explanation for these varied results in relation to the possible genetic association between impulsivity and alcohol use is that when selecting for a particular phenotype, in this case differential levels of home cage drinking and preference for alcohol solutions over water, the phenotype of interest might in the first instance be determined by a particular aggregate of secondary phenotypes (reactivity to novelty, taste aversion, etc) that could vary between the different parental strains that are bi-directionally bred over generations, with the subsequent selection of the accompanying genes associated with those selected phenotypes (and genes) [36].

A 4-week period of intermittent access to alcohol had no effect on impulsivity in P or in NP rats. As expected, the NP line did not voluntarily drink alcohol (<1g/kg x 24h) and so it was to be expected that their level of impulsivity did not change as a consequence of their modest alcohol intake. However, nor did alcohol have any effect on impulsive performance of P rats in the 5-CSRTT and this is in accordance with other studies reporting no differences in choice impulsivity in rats [37] or motor impulsivity in mice [38] after extended access to alcohol. Several factors can account for this lack of effect of alcohol in our experiment. Firstly, the total amount of alcohol consumed by the P rats, while significant, was moderate (P rats consumed a mean total of 34.3 ± 2.3 g/kg alcohol, while NP rats only 2.92 ± 0.64 g/kg) and possibly insufficient to influence impulsivity in contrast to other studies in which higher amounts of alcohol and longer treatment durations were used [39,40]. Nevertheless, our results are in accordance with those of Mejia-Toiber et al [37] in which adult rats exposed to chronic intermittent ethanol did not differ from saline treated rats in their impulsivity levels in a delay discounting task. Secondly, in the present experiment impulsivity may have been assessed outside the critical withdrawal period: in the current study a total of 22 days passed between the end of the intermittent two bottle choice procedure and the post-alcohol long ITI testing, a period in which the effects of alcohol in increasing premature responding could have been mitigated. However, this is not in accordance with previous results showing increased motor impulsivity (in the 5-CSRTT and in the 5 choice continuous performance task) in rats after repeated cycles of alcohol intoxication and withdrawal that persisted even after 5 weeks of abstinence [39,40]. The current results suggest that disruptive effects on impulsivity of chronic alcohol exposure in adult rats may only be seen with greater degrees of alcohol intake.

In contrast to the lack of group differences regarding the effects of alcohol exposure described above, we did find robust differences between P and NP rats in measures of motivation, specifically magazine latencies and in the time spent in the food magazine but not in attentional variables such as accuracy of responding and omissions. Thus, P rats showed consistently faster latencies to collect the food reward after a correct response and also spent longer time responding in the food magazine compared with NP rats. These observations suggest P rats were more highly motivated than NP rats to forage and consume the food reward. Consistent with these findings P rats have also been found to display a higher preference for sucrose [41] and saccharin [42] compared with NP rats.

When tested in the Pavlovian conditioned approach task NP rats developed a sign-tracking (lever-press) response. However, this was absent in the P rats, who, nevertheless, showed high levels of goal-tracking behaviour, manifesting perseverative nose-poking in the food magazine during the periods in which the conditioned CS lever was inserted into the chamber, signalling the proximal delivery of the reinforcer, a result that positively correlated with time spent nose-poking in the magazine in the 5-CSRTT. The full significance of these results is still unclear but goal-tracking trait may be co-selected during the bi-directional breeding of the alcohol preferring phenotype, as is the case for rats selectively bred based on their high or low reactivity to a novel environment, the bred high-responder (bHR) and bred low-responder (bLR) rats, which similarly display marked differences in goal- and sign-tracking [25]. The literature regarding the relationship between impulsivity and sign-/goal-tracking behaviour is varied, and seems to depend on the dimension of impulsivity being measured. Tomie et al [43] initially reported a positive association between choice impulsivity, evaluated in a delay discounting task, and the acquisition of autoshaping in rats [43] but this result has not been replicated in two successive studies: Flagel et al. [25] found the opposite result when assessing bHR and bLR rats in a delay discounting task, reporting that the bHR rats, which also show high sign-tracking behaviour, where less impulsive than the bLR rats, which are goal-trackers. In contrast, the same study found higher motor impulsivity, evaluated in a DRL task, in bHR rats, in comparison with bLR rats, a result later replicated by Lovic et al [26], in which sign-tracking was positively associated with motor impulsivity in a two-choice serial reaction time task and in a DRL task. However, the later study seemed to suggest the opposite relationship regarding choice impulsivity, where goal-trackers showed higher levels of impulsivity in the longer delay option in comparison with sign-trackers. Although these two later studies showed a positive association between motor impulsivity and the acquisition of sign-tracking response, we failed to see any such association between high or low impulsive rats in the 5-CSRTT and Pavlovian autoshaping [31]. In the present experiment, we also failed to find any correlation between motor impulsivity in the 5-CSRTT and goal- or sign-tracking behaviour. Conversely, our findings do support a dichotomy between goal- and sign-tracking responses, which were negatively inter-related, and also report for the first time a significant relationship between food-magazine perseveration in the 5-CSRTT and goal-tracking responses in a Pavlovian conditioned approach task, as previously reported [44].

The high goal-tracking responses seen in P rats in the present investigation seem to be a counterintuitive finding according to the sign-tracking model of alcohol abuse proposed by Tomie and colleagues [45]. An alternative explanation could be that the door/panel which gives access to the food reinforcer in the magazine of the 5-CSRTT or even the magazine itself in the Pavlovian approach task could represent the “cocktail glass” CS alluded to by Tomie, insofar as the magazine is the receptacle in which the reward is consumed by the rat. However, a similar argument could be made about goal-tracking in general; it is controlled by stimuli in the immediate vicinity of the food reinforcer. Our observations suggest, therefore, that high levels of goal-tracking of a food reinforcer in an appetitive Pavlovian approach task might predict elevated levels of consumption and preference for alcohol, but establishing a truly causal link between the two will require further study.

To conclude, high levels of genetically determined alcohol preference and consumption is not associated with increased impulsivity in the 5-CSRTT. Thus, motor impulsivity in this task predicts higher rates of cocaine [28] and nicotine self-administration [13] but not heroin [14] or alcohol self-administration. These results suggest that 5-CSRTT impulsivity and vulnerability to addiction may hold only for psychostimulant substances but may not always generalise to opioids or alcohol. Our findings further demonstrate that excessive food-oriented behaviour during performance on the 5-CSRTT and an appetitive Pavlovian conditioning task is associated with increased preference and consumption of alcohol, implying that shared mechanisms may underlie goal-tracking to food incentives and alcohol intake.

## Supporting Information

S1 Fig Pavlovian conditioned approach. Alcohol-preferring rats (P, closed squares) and alcohol-non-preferring rats (NP, open circles). Data are means ± SE of probability of lever press (a), probability of nose-poke (b), latency of lever press (c) response bias [(number of lever presses-number of magazine nose-pokes)/(number of lever presses + number of magazine nose-pokes)] (d), and number of nose-pokes from trials 1 to 25 during the first session (e). *Statistics*: repeated measures ANOVA for each line were performed followed by paired samples t tests comparing each of the sessions with session 1 for graphs A-D, and comparing trial 25 vs trial 1 in graph E. *Results*: Probability of lever press: P rats did not significantly increase their probability of lever press across sessions (lack of session effect (F (4,56) = 1.662, n.s), but P rats showed an increase in the probability of lever press across sessions (F (4,48) = 6.112, p<0.01, ε = .653). Probability of nose-poking: No significant session effect in either line. Latency lever press: both lines showed a reduction in latencies to lever press across sessions (P rats F(4,56) = 3.86, p<0.01, NP rats F (4,48) = 11.638 p<0.001). Paired samples t test showed that this decreased in latencies occurred earlier (from session 2) in NP rats as compared with P rats (from session 4). Response bias: P rats performed more nose-poke behaviour than lever presses and this did not change over time (no significant session effect (F (4,56) = 2.192, n.s), whereas NP rats progressively increase their lever press behaviour in comparison with their nose-poke behaviour (significant effect of session: F (4,48) = 4.049, p<0.05, ε = .612). Nose-pokes in session 1: P rats showed an increase in nose-poking behaviour in the magazine within the first session of Pavlovian conditioned approach (significant effect of session (F(1,14) = 13.808, p<0.01), whereas NP rats showed stable levels of nose-poking throughout the session (no session effect: F(1,12) = 0.013, n.s.).
(PDF)

## References

[1] Schulsinger F, Knop J, Goodwin DW, Teasdale TW, Mikkelsen U. A prospective study of young men at high risk for alcoholism. Social and psychological characteristics. Arch Gen Psychiatry. 1986;43: 755–760. 3729670 10.1001/archpsyc.1986.01800080041006

[2] Petry NM, Kirby KN, Kranzler HR. Effects of gender and family history of alcohol dependence on a behavioral task of impulsivity in healthy subjects. J Stud Alcohol. 2002;63: 83–90. 11925063

[3] Ernst M, Luckenbaugh DA, Moolchan ET, Leff MK, Allen R, Eshel N, et al Behavioral predictors of substance-use initiation in adolescents with and without attention-deficit/hyperactivity disorder. Pediatrics. 2006;117: 2030–2039. 10.1542/peds.2005-0704 16740845

[4] Bell RL, Rodd ZA, Lumeng L, Murphy JM, McBride WJ. The alcohol-preferring P rat and animal models of excessive alcohol drinking. Addict Biol. 2006;11: 270–288. 10.1111/j.1369-1600.2005.00029.x 16961759

[5] Li TK, Lumeng L, Doolittle DP. Selective breeding for alcohol preference and associated responses. Behav Genet. 1993;23: 163–170. 8099788 10.1007/BF01067421

[6] Verdejo-García A, Lawrence AJ, Clark L. Impulsivity as a vulnerability marker for substance-use disorders: review of findings from high-risk research, problem gamblers and genetic association studies. Neurosci Biobehav Rev. 2008;32: 777–810. 10.1016/j.neubiorev.2007.11.003 18295884

[7] Petry NM. Delay discounting of money and alcohol in actively using alcoholics, currently abstinent alcoholics, and controls. Psychopharmacology (Berl). 2001;154: 243–250. 11351931 10.1007/s002130000638

[8] Sanchez-Roige S, Baro V, Trick L, Peña-Oliver Y, Stephens DN, Duka T. Exaggerated waiting impulsivity associated with human binge drinking, and high alcohol consumption in mice. Neuropsychopharmacol Off Publ Am Coll Neuropsychopharmacol. 2014;39: 2919–2927. 10.1038/npp.2014.151 PMC4229569 24947901

[9] Loos M, Staal J, Smit AB, De Vries TJ, Spijker S. Enhanced alcohol self-administration and reinstatement in a highly impulsive, inattentive recombinant inbred mouse strain. Front Behav Neurosci. 2013;7: 151 10.3389/fnbeh.2013.00151 24198771 PMC3812782

[10] Wilhelm CJ, Reeves JM, Phillips TJ, Mitchell SH. Mouse lines selected for alcohol consumption differ on certain measures of impulsivity. Alcohol Clin Exp Res. 2007;31: 1839–1845. 10.1111/j.1530-0277.2007.00508.x 17850219

[11] Mole TB, Irvine MA, Worbe Y, Collins P, Mitchell SP, Bolton S, et al. Impulsivity in disorders of food and drug misuse. Psychol Med. 2014; 1–12. 10.1017/S0033291714001834 PMC4998952 25118940

[12] Belin D, Mar AC, Dalley JW, Robbins TW, Everitt BJ. High impulsivity predicts the switch to compulsive cocaine-taking. Science. 2008;320: 1352–1355. 10.1126/science.1158136 18535246 PMC2478705

[13] Diergaarde L, Pattij T, Poortvliet I, Hogenboom F, de Vries W, Schoffelmeer ANM, et al Impulsive choice and impulsive action predict vulnerability to distinct stages of nicotine seeking in rats. Biol Psychiatry. 2008;63: 301–308. 10.1016/j.biopsych.2007.07.011 17884016

[14] McNamara R, Dalley JW, Robbins TW, Everitt BJ, Belin D. Trait-like impulsivity does not predict escalation of heroin self-administration in the rat. Psychopharmacology (Berl). 2010;212: 453–464. 10.1007/s00213-010-1974-9 20689939

[15] Steinmetz JE, Blankenship MR, Green JT, Smith GB, Finn PR. Evaluation of behavioral disinhibition in P/NP and HAD1/LAD1 rats. Prog Neuropsychopharmacol Biol Psychiatry. 2000;24: 1025–1039. 11041542 10.1016/s0278-5846(00)00122-6

[16] McMillen BA, Means LW, Matthews JN. Comparison of the alcohol-preferring P rat to the Wistar rat in behavioral tests of impulsivity and anxiety. Physiol Behav. 1998;63: 371–375. 9469729 10.1016/s0031-9384(97)00442-3

[17] Oberlin BG, Grahame NJ. High-alcohol preferring mice are more impulsive than low-alcohol preferring mice as measured in the delay discounting task. Alcohol Clin Exp Res. 2009;33: 1294–1303. 10.1111/j.1530-0277.2009.00955.x 19389183 PMC2872785

[18] Beckwith SW, Czachowski CL. Increased delay discounting tracks with a high ethanol-seeking phenotype and subsequent ethanol seeking but not consumption. Alcohol Clin Exp Res. 2014;38: 2607–2614. 10.1111/acer.12523 25335779 PMC4251872

[19] Wilhelm CJ, Mitchell SH. Rats bred for high alcohol drinking are more sensitive to delayed and probabilistic outcomes. Genes Brain Behav. 2008;7: 705–713. 10.1111/j.1601-183X.2008.00406.x 18518928 PMC2789313

[20] Wilhelm CJ, Mitchell SH. Acute ethanol does not always affect delay discounting in rats selected to prefer or avoid ethanol. Alcohol Alcohol Oxf Oxfs. 2012;47: 518–524. 10.1093/alcalc/ags059 22645038 PMC3500854

[21] Diergaarde L, van Mourik Y, Pattij T, Schoffelmeer ANM, De Vries TJ. Poor impulse control predicts inelastic demand for nicotine but not alcohol in rats. Addict Biol. 2012;17: 576–587. 10.1111/j.1369-1600.2011.00376.x 21966927

[22] Flagel SB, Akil H, Robinson TE. Individual differences in the attribution of incentive salience to reward-related cues: Implications for addiction. Neuropharmacology. 2009;56 Suppl 1: 139–148. 10.1016/j.neuropharm.2008.06.027 18619474 PMC2635343

[23] Saunders BT, Robinson TE. A cocaine cue acts as an incentive stimulus in some but not others: implications for addiction. Biol Psychiatry. 2010;67: 730–736. 10.1016/j.biopsych.2009.11.015 20045508 PMC2849872

[24] Stoffel EC, Cunningham KA. The relationship between the locomotor response to a novel environment and behavioral disinhibition in rats. Drug Alcohol Depend. 2008;92: 69–78. 10.1016/j.drugalcdep.2007.06.012 17997051

[25] Flagel SB, Robinson TE, Clark JJ, Clinton SM, Watson SJ, Seeman P, et al An animal model of genetic vulnerability to behavioral disinhibition and responsiveness to reward-related cues: implications for addiction. Neuropsychopharmacol Off Publ Am Coll Neuropsychopharmacol. 2010;35: 388–400. 10.1038/npp.2009.142 PMC2794950 19794408

[26] Lovic V, Saunders BT, Yager LM, Robinson TE. Rats prone to attribute incentive salience to reward cues are also prone to impulsive action. Behav Brain Res. 2011;223: 255–261. 10.1016/j.bbr.2011.04.006 21507334 PMC3119757

[27] Flagel SB, Waselus M, Clinton SM, Watson SJ, Akil H. Antecedents and consequences of drug abuse in rats selectively bred for high and low response to novelty. Neuropharmacology. 2014;76 Pt B: 425–436. 10.1016/j.neuropharm.2013.04.033 23639434 PMC3766490

[28] Dalley JW, Fryer TD, Brichard L, Robinson ESJ, Theobald DEH, Lääne K, et al Nucleus accumbens D2/3 receptors predict trait impulsivity and cocaine reinforcement. Science. 2007;315: 1267–1270. 10.1126/science.1137073 17332411 PMC1892797

[29] Fitzpatrick CJ, Gopalakrishnan S, Cogan ES, Yager LM, Meyer PJ, Lovic V, et al Variation in the form of Pavlovian conditioned approach behavior among outbred male Sprague-Dawley rats from different vendors and colonies: sign-tracking vs. goal-tracking. PloS One. 2013;8: e75042 10.1371/journal.pone.0075042 24098363 PMC3787975

[30] Whelan R, Watts R, Orr CA, Althoff RR, Artiges E, Banaschewski T, et al Neuropsychosocial profiles of current and future adolescent alcohol misusers. Nature. 2014;512: 185–189. 10.1038/nature13402 25043041 PMC4486207

[31] Robinson ESJ, Eagle DM, Economidou D, Theobald DEH, Mar AC, Murphy ER, et al Behavioural characterisation of high impulsivity on the 5-choice serial reaction time task: specific deficits in “waiting” versus “stopping.” Behav Brain Res. 2009;196: 310–316. 10.1016/j.bbr.2008.09.021 18940201

[32] Evenden JL. Varieties of impulsivity. Psychopharmacology (Berl). 1999;146: 348–361. 10550486 10.1007/pl00005481

[33] Eagle DM, Baunez C. Is there an inhibitory-response-control system in the rat? Evidence from anatomical and pharmacological studies of behavioral inhibition. Neurosci Biobehav Rev. 2010;34: 50–72. 10.1016/j.neubiorev.2009.07.003 19615404 PMC2789250

[34] Richards JB, Lloyd DR, Kuehlewind B, Militello L, Paredez M, Solberg Woods L, et al Strong genetic influences on measures of behavioral-regulation among inbred rat strains. Genes Brain Behav. 2013;12: 490–502. 10.1111/gbb.12050 23710681 PMC4425401

[35] Broos N, Schmaal L, Wiskerke J, Kostelijk L, Lam T, Stoop N, et al The relationship between impulsive choice and impulsive action: a cross-species translational study. PloS One. 2012;7: e36781 10.1371/journal.pone.0036781 22574225 PMC3344935

[36] Crabbe JC, Phillips TJ, Belknap JK. The complexity of alcohol drinking: studies in rodent genetic models. Behav Genet. 2010;40: 737–750. 10.1007/s10519-010-9371-z 20552264 PMC3330823

[37] Mejia-Toiber J, Boutros N, Markou A, Semenova S. Impulsive choice and anxiety-like behavior in adult rats exposed to chronic intermittent ethanol during adolescence and adulthood. Behav Brain Res. 2014;266: 19–28. 10.1016/j.bbr.2014.02.019 24566059 PMC4005391

[38] Walker SE, Peña-Oliver Y, Stephens DN. Learning not to be impulsive: disruption by experience of alcohol withdrawal. Psychopharmacology (Berl). 2011;217: 433–442. 10.1007/s00213-011-2298-0 21509502

[39] Irimia C, Tuong RN, Quach T, Parsons LH. Impaired response inhibition in the rat 5 choice continuous performance task during protracted abstinence from chronic alcohol consumption. PloS One. 2014;9: e109948 10.1371/journal.pone.0109948 25333392 PMC4198178

[40] Irimia C, Wiskerke J, Natividad LA, Polis IY, de Vries TJ, Pattij T, et al. Increased impulsivity in rats as a result of repeated cycles of alcohol intoxication and abstinence. Addict Biol. 2013; 10.1111/adb.12119 PMC4061283 24341858

[41] Stewart RB, Russell RN, Lumeng L, Li TK, Murphy JM. Consumption of sweet, salty, sour, and bitter solutions by selectively bred alcohol-preferring and alcohol-nonpreferring lines of rats. Alcohol Clin Exp Res. 1994;18: 375–381. 8048741 10.1111/j.1530-0277.1994.tb00028.x

[42] Sinclair JD, Kampov-Polevoy A, Stewart R, Li TK. Taste preferences in rat lines selected for low and high alcohol consumption. Alcohol Fayettev N. 1992;9: 155–160. 10.1016/0741-8329(92)90027-8 1599627

[43] Tomie A, Aguado AS, Pohorecky LA, Benjamin D. Ethanol induces impulsive-like responding in a delay-of-reward operant choice procedure: impulsivity predicts autoshaping. Psychopharmacology (Berl). 1998;139: 376–382. 9809858 10.1007/s002130050728

[44] Murray JE, Dilleen R, Pelloux Y, Economidou D, Dalley JW, Belin D, et al Increased impulsivity retards the transition to dorsolateral striatal dopamine control of cocaine seeking. Biol Psychiatry. 2014;76: 15–22. 10.1016/j.biopsych.2013.09.011 24157338 PMC4064115

[45] Tomie A, Sharma N. Pavlovian sign-tracking model of alcohol abuse. Curr Drug Abuse Rev. 2013;6: 201–219. 24694108 10.2174/18744737113069990023

