# Correction: Alcohol-Preferring Rats Show Goal Oriented Behaviour to Food Incentives but Are Neither Sign-Trackers Nor Impulsive

**DOI:** 10.1371/journal.pone.0134198

**Published:** 2015-07-24

**Authors:** Yolanda Peña-Oliver, Chiara Giuliano, Daina Economidou, Charles R. Goodlett, Trevor W. Robbins, Jeffrey W. Dalley, Barry J. Everitt

There are errors in the Funding section. The correct funding information is as follows: The present study was funded by the Wellcome Trust and the Medical Research Council Programme (MRC Ref: G1002231 awarded to BJE, JWD, TWR, Wellcome Trust Ref: 093875/Z/10/Z), and the R24 Alcohol Research Resource Award grant (R24 AA015512) from NIAAA. The funders had no role in study design, data collection and analysis, decision to publish, or preparation of the manuscript.
