# Supplementary material for: Alcohol-Preferring Rats Show Goal Oriented Behaviour to Food Incentives but Are Neither Sign-Trackers Nor Impulsive
Source: PLoS One. 2015 Jun 22;10(6):e0131016. doi: 10.1371/journal.pone.0131016 (PMC4476783; doi:10.1371/journal.pone.0131016)

Supporting figure 1. Pavlovian conditioned approach

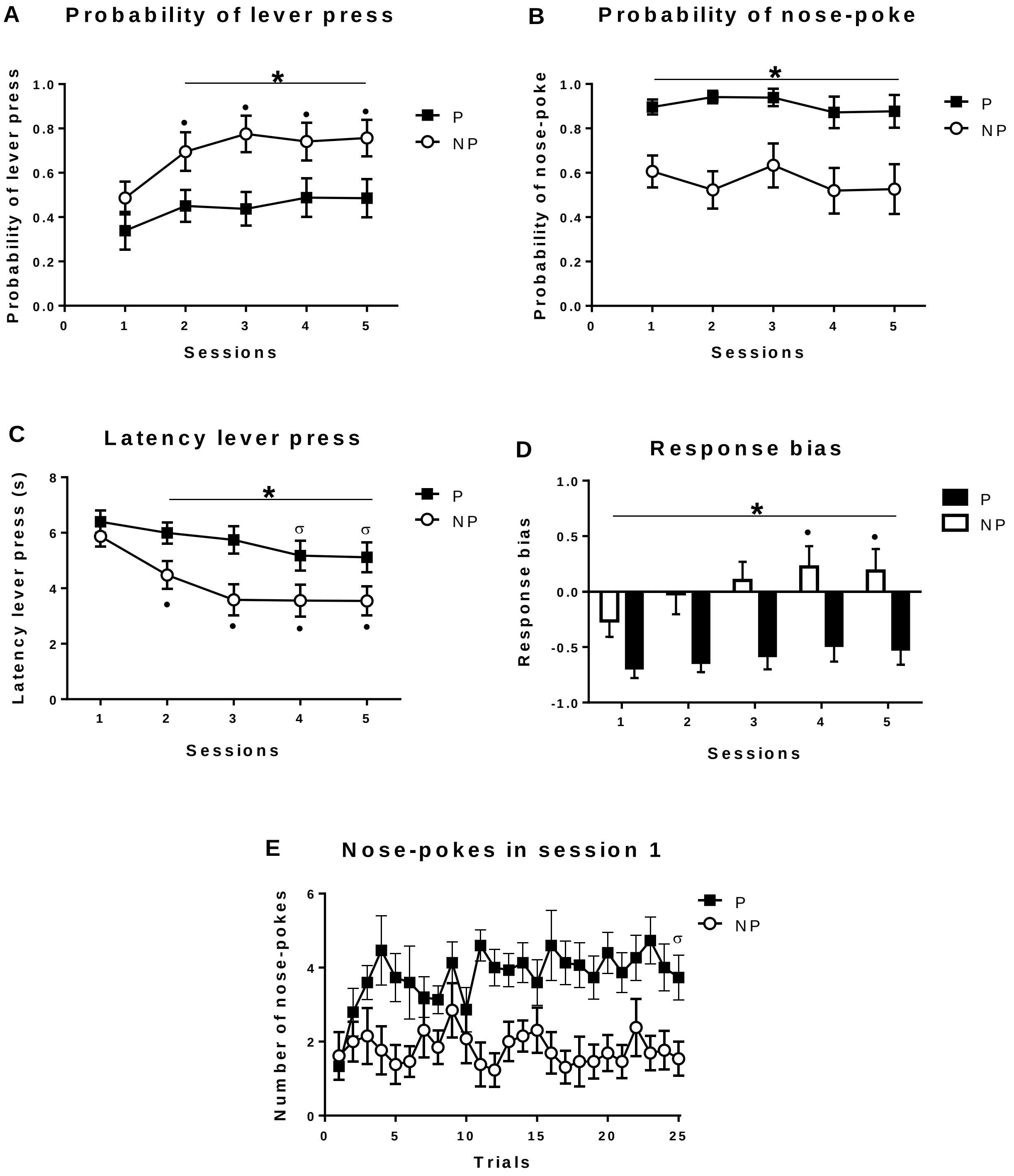

Supplement: S1 Fig — Alcohol-preferring rats (P, closed squares) and alcohol-non-preferring rats (NP, open circles). Data are means ± SE of probability of lever press (a), probability of nose-poke (b), latency of lever press (c) response bias [(number of lever presses-number of magazine nose-pokes)/(number of lever presses + number of magazine nose-pokes)] (d), and number of nose-pokes from trials 1 to 25 during the first session (e). Statistics: repeated measures ANOVA for each line were performed followed by paired samples t tests comparing each of the sessions with session 1 for graphs A-D, and comparing trial 25 vs trial 1 in graph E. Results: Probability of lever press: P rats did not significantly increase their probability of lever press across sessions (lack of session effect (F (4,56) = 1.662, n.s), but P rats showed an increase in the probability of lever press across sessions (F (4,48) = 6.112, p<0.01, ε = .653). Probability of nose-poking: No significant session effect in either line. Latency lever press: both lines showed a reduction in latencies to lever press across sessions (P rats F(4,56) = 3.86, p<0.01, NP rats F (4,48) = 11.638 p<0.001). Paired samples t test showed that this decreased in latencies occurred earlier (from session 2) in NP rats as compared with P rats (from session 4). Response bias: P rats performed more nose-poke behaviour than lever presses and this did not change over time (no significant session effect (F (4,56) = 2.192, n.s), whereas NP rats progressively increase their lever press behaviour in comparison with their nose-poke behaviour (significant effect of session: F (4,48) = 4.049, p<0.05, ε = .612). Nose-pokes in session 1: P rats showed an increase in nose-poking behaviour in the magazine within the first session of Pavlovian conditioned approach (significant effect of session (F(1,14) = 13.808, p<0.01), whereas NP rats showed stable levels of nose-poking throughout the session (no session effect: F(1,12) = 0.013, n.s.). (PDF) [file pone.0131016.s001.pdf]
